# Dataset of proteins mapped on HepG2 cells and those differentially abundant after expression of the dengue non-structural 1 protein

**DOI:** 10.1016/j.dib.2016.11.083

**Published:** 2016-12-06

**Authors:** Kíssila Rabelo, Monique R.O. Trugilho, Simone M. Costa, André T.S. Ferreira, Paulo C. Carvalho, Jonas Perales, Ada M.B. Alves

**Affiliations:** a Laboratory of Biotechnology and Physiology of Viral Infections, Oswaldo Cruz Institute, Fiocruz, Rio de Janeiro, Brazil; b Laboratory of Toxinology, Oswaldo Cruz Institute, Fiocruz, Rio de Janeiro, Brazil; c Laboratory for Proteomics and Protein Engineering, Carlos Chagas Institute, Fiocruz, Paraná, Brazil

**Keywords:** Mass spectrometry, NS1, DENV, HepG2 cells

## Abstract

The data supplied in this article are related to the research article entitled “The effect of the dengue non-structural 1 protein expression over the HepG2 cell proteins in a proteomic approach” (K. Rabelo, M.R. Trugillo, S.M. Costa, B.A. Pereira, O.C. Moreira, A.T. Ferreira et al., 2016) [1]. The present article provides the inventory of peptides and proteins mapped in a hepatocyte cell line (HepG2) by mass spectrometry in the presence of the non-structural protein 1 (NS1) of Dengue 2 virus (DENV2). Cells were transfected with pcENS1 plasmid, which encodes the DENV2 NS1 protein, or the controls pcDNA3 (negative control) or pMAXGFP, encoding the green fluorescent protein (GFP), a protein unrelated to dengue. Differentially abundant protein lists were obtained by comparing cells transfected with pcENS1 and controls.

**Specifications Table**

**Table:** 

Subject area	*Biology*
More specific subject area	*Proteomics, Virology*
Type of data	*Table*
How data was acquired	*Mass spectrometry*
Data format	*Raw and analyzed*
Experimental factors	*HepG2 cells were transfected with plasmids expressing different proteins, lysed, trypsinized and submitted to Orbitrap*
Experimental features	*All samples were analyzed LTQ-Orbitrap XL mass spectrometer*
Data source location	*Oswaldo Cruz Foundation, Brazil*
Data accessibility	*Within this article*

**Value of the data**

• These data describe the use of quantitative mass spectrometry-based proteomic experiments to assess the biological significance of cell alterations caused by DENV NS1 protein.
• 4756 proteins were mapped and we identify 41 or 81 differentially abundant proteins in the presence of NS1, comparing to controls.
• The data open new perspectives to identify the molecular mechanisms involving DENV NS1 protein in infected cells.

## 1 Data

HepG2 cells were transfected with the plasmids: pcENS1, pcDNA3 and pMAXGFP. To produce accurate data, we used three independent experimental biological replicates and samples were submitted to LTQ-Orbitrap XL (Thermo Scientific). Data analysis, using the PatternLab for Proteomics software, identified 14,138 peptides which mapped to 4756 proteins, from all conditions (HepG2 transfected with the three different plasmids and non-transfected cells) (Supplementary Table S1a–h). Applying the maximum parsimony principle we found 2314 proteins (Supplementary Table S1g). Using the Tfold module we generate the differential abundance distribution when comparing: non-transfected HepG2 x cell transfected with pcDNA3 (Table 1); HepG2 transfected with pcDNA3 x pcENS1 (Table 2) and cells transfected with pMAGFP x pcENS1 (Table 3)[1].

## 2 Experimental design, materials and methods

### 2.1 Cell culture

HepG2 cells (ATCC) were cultivated in Dulbecco׳s modified Eagle׳s medium (DMEM) (SIGMA) supplemented with 10% fetal bovine serum (FBS) (Invitrogen). Cells were maintained at 37° C and under humid atmosphere with 5% CO_2_. In all experiments, cells were transfected between 86 and 89 cell passages with 70–80% confluence.

### 2.2 Plasmids

The recombinant plasmid pcENS1 was previously constructed in our laboratory [2], using the pcDNA3 mammalian expression vector (Invitrogen). It contains the sequence of 63 nucleotides that encodes 21 amino acids from the C-terminal portion of the DENV2 envelope (E) protein and the full length DENV2 *ns1* gene. The vector pcDNA3 was used as a negative control, while the plasmid pMAXGFP (Amaxa), which encodes the green fluorescent protein (GFP) from *Pontellina plumata* copepod, was used as a control for expression of a DENV non-related protein.

### 2.3 Transfection

Transfection was performed by nucleofection with the Nucleofector V™ kit (Amaxa), according to manufacturer׳s recommendation. Briefly, HepG2 cells were seeded on 75 cm^2^ bottles, harvested after 4 or 5 days with the aid of cell scrapes in 3 ml of CMF solution (8 g/L of NaCl; 0.4 g/L of KCl; 0.1 g/L of Na_2_SO_4_; 0.39 g/L of Na_2_HPO_4_.12H_2_O; 0.15 g/L of KH_2_PO_4_; 1.1 g/L of glucose; 0.0025 g/L of phenol red, pH 7.4), centrifuged at 500 g for 5 min and suspended in the nucleofection solution (Amaxa). Cell suspension with 5 μg of DNA plasmids (10^6^ cells/100 μl/cuvettes) was submitted to an electric shock in the Nucleofector 6 equipment (Amaxa), using the T-28 program. Nine cuvettes were used for each sample (pcDNA3, pcENS1 or pMAXGFP). After shock, cells received 500 μL of DMEM with 10% FBS and were immediately transferred to microcentrifuge tubes containing another 500 μL DMEM with 10% FBS. Cells were seeded on 25 cm^2^ flasks, incubated in humid atmosphere with 5% CO_2_ at 37 °C for 24 h.

### 2.4 Proteomic sample preparation

Cells were centrifuged at 500 g for 10 min and suspended in 50 mM ammonium bicarbonate buffer containing 0.2% of RapiGest™ SF (Waters). The protein concentration was determined using Qubit 2.0^®^ kit (Invitrogen) following the manufacturer׳s instructions. A total of 50 μg protein was used for each sample. Samples were treated with 5 μL of 100 mM dithiothreitol for reduction, incubated for 3 h at 37 °C. After reaching room temperature, samples were alkylated with 5 μL of 400 mM iodoacetamide for 15 min, in the dark. Trypsin (Promega) was added in the ratio 1:50 enzyme/substrate and digestion was performed for 20 h, at 37 °C. The reaction was stopped after adding formic acid to final concentration of 1%. Aliquots from this digestion were desalted by using POROS R2 C8–18 resin (Invitrogen), packaged in micropipette tips (Millipore) and equilibrated in TFA 1%. After washing with 0.1% TFA, peptides were eluted in 0.1% TFA with 70% acetonitrile and completely dried in the vacuum centrifuge.

### 2.5 Isoelectric focalization of peptides (OFFGEL)

Twenty five micrograms of peptides were solubilized in 1.8 mL of 0.01% ampholytes (OFFGEL buffer pH 3–10) containing 4% (*v/v*) glycerol and was submitted to the 3100 OFFGEL Fractionator with the OFFGEL Low Res Kit pH 3–10 (Agilent Technologies) immobilized pH gradient (IPG) DryStrips, following the Agilent׳s instructions. The peptides were also separated according to the manufacturer׳s instruction and optimized as described in Hubner et al. [3]. Twelve well fractionations were focused for 20 kV with a maximum current of 50 mA and power of 200 mW for 24 h. Each fraction was separately desalted as previously described and suspended in 40 μL of 1% formic acid. All fractions were analyzed on 10 cm reversed phase (RP) column coupled to an LTQ-Orbitrap XL mass spectrometer.

### 2.6 Liquid chromatography-tandem mass spectrometry (LC-MS/MS) analysis

Desalted peptides fractions were loaded separately onto a 10 cm RP column coupled to the mass spectrometer by using a Proxeon easy-nLC-System (Thermo Scientific Easy-nLC II). Four microliters were initially applied to a 2 cm long (100 µm internal diameter) trap column packed with 5 µm, 200 Å Magic C18 AQ matrix (Michrom Bioresources) followed by separation on a 10 cm long (75 µm internal diameter) separation column packed with the same matrix directly on a self-pack 5–15 µm Tip empty column (New Objective). Samples were loaded onto the trap column at 2 μL/min while chromatographic separation occurred at 200 nL/min. Mobile phase A consisted of 0.1% formic acid in water while mobile phase B consisted of 0.1% formic acid in acetonitrile. Peptides were eluted with a gradient of 2–40% of B over 32 min followed by up to 80% B in 4 min, maintaining at this concentration for 2 min more, before column equilibration. The HPLC system was coupled to the LTQ-Orbitrap XL via a nanoscale LC interface (Thermo Scientific). Source voltage was set to 1.9 kV, the temperature of heated capillary was set to 200 °C and tube lens voltage to 48 and 100 V, respectively. The target precursor specters were acquired in ion trap full scan MS with 60,000 while FWHM full AGC target was set to 500,000. MS1 spectra were acquired on the Orbitrap analyzer (300–1700 m/z) at a 60,000 resolution (for m/z 445.1200). For each spectrum, the 10 most intense ions were submitted to CID fragmentation (minimum signal required of 10,000; isolation width of 2.5; normalized collision energy of 35.0; activation Q of 0.25 and activation time of 30 s, followed by MS2 acquisition on the linear trap quadrupole analyzer. Dynamic exclusion option was enabled and set with the following values for each parameter: repeat count=1; repeat duration=30 s; exclusion list size=500; exclusion duration=45 s and exclusion mass width=10 ppm. Data were acquired in technical triplicates using the Xcalibur software (version 2.0.7).

### 2.7 Protein identification

The raw data files were processed and quantified using PatternLab for Proteomics software v 3.2 [4] (available at: http://max.ioc.fiocruz.br/mtrugilho/RabeloK2016/). Peptide sequence matching (PSM) was performed using the Comet algorithm [5] against the UniProt database (http://www.uniprot.org/) with human proteins entries downloaded January 2015, plus a FASTA file containing Dengue virus and GFP sequences, retrieved from the NCBI database. A target-reverse strategy was employed for increased confidence in protein identifications [6]. The search considered tryptic and semi-tryptic peptide candidates. The cysteine carbamidomethylation and oxidation of methionine were considered as fixed and variable modifications, respectively. The Comet search engine considered a precursor mass tolerance of 40 ppm and bins of 1.0005 for the MS/MS. The validity of the peptide spectrum matches were assessed using PatternLab׳s Search Engine Processor (SEPro) module [7]. Briefly, identifications were grouped by charge state (+2 and >+3) and then by tryptic status (i.e., tryptic or semi-tryptic), resulting in four distinct subgroups. For each result, the XCorr, DeltaCN and Secondary Score values were used to generate a Bayesian discriminator. SEPro then automatically established a cutoff score to accept a false-discovery rate (FDR) of 1% based on the number of decoys, independently performed on each data subset, resulting in a false-positive rate that was independent of tryptic status or charge state [7]. Additionally, a minimum sequence length of 6 amino acid residues was required. Then, only PSMs with less than 5 ppm were considered to compose a final list of proteins supported by at least three independent evidences (e.g., identification of a peptide in different charge states, modified and non-modified version of the same peptide, or different peptides). All identification results are reported with less than 1% FDR, both peptide and protein level, by PatternLab׳s SEPro module. Spectral counting for estimation of protein copy number was accomplished using the normalized spectral abundance factor (NSAF) [8]. These conditions generate 14,138 peptides which mapped to 4756 proteins, from all samples (HepG2 transfected with the three different plasmids and non-transfected cells) (Supplementary Table S1a–h). Applying the maximum parsimony principle we found 2314 proteins (Supplementary Table S1g). Differentially abundant proteins were pinpointed (Table 2a–c) using PatternLab׳s TFold module with a Benjamini–Hochberg *q*-value of 0.05 [9].

## Figures and Tables

**Table 1:** List of 54 differentially abundant proteins for statistics between HepG2 x pcDNA3.

**Locus**	**Fold Change**	***p*Value**	**Signal+(pcDNA3)**	**Signal-(HepG2)**	**Description**
sp|P20674|COX5A_HUMAN	7.52	0.04600	1.29E-03	1.72E-04	Cytochrome *c* oxidase subunit 5A, mitochondrial OS=Homo sapiens GN=COX5A PE=1 SV=2
sp|P39656|OST48_HUMAN	6.45	0.00016	9.18E-04	1.42E-04	Dolichyl-diphosphooligosaccharide--protein glycosyltransferase 48 kDa subunit OS=Homo sapiens GN=DDOST PE=1 SV=4
sp|Q71UI9|H2AV_HUMAN	4.20	0.02071	5.62E-03	1.34E-03	Histone H2A.V OS=Homo sapiens GN=H2AFV PE=1 SV=3
sp|P0C0S5|H2AZ_HUMAN	4.18	0.02251	5.58E-03	1.34E-03	Histone H2A.Z OS=Homo sapiens GN=H2AFZ PE=1 SV=2
sp|P02768|ALBU_HUMAN	3.40	0.01591	6.34E-04	1.87E-04	Serum albumin OS=Homo sapiens GN=ALB PE=1 SV=2
tr|B4DLR8|B4DLR8_HUMAN	3.32	0.01500	2.00E-03	6.04E-04	NAD(P)H dehydrogenase [quinone] 1 OS=Homo sapiens GN=NQO1 PE=1 SV=1
sp|P15559|NQO1_HUMAN	3.27	0.01639	1.45E-03	4.45E-04	NAD(P)H dehydrogenase [quinone] 1 OS=Homo sapiens GN=NQO1 PE=1 SV=1: tr|H3BNV2|H3BNV2_HUMAN NAD(P)H dehydrogenase [quinone] 1 OS=Homo sapiens GN=NQO1 PE=1 SV=1
sp|P13073|COX41_HUMAN	3.16	0.04534	5.81E-04	1.84E-04	Cytochrome *c* oxidase subunit 4 isoform 1, mitochondrial OS=Homo sapiens GN=COX4I1 PE=1 SV=1: tr|H3BPG0|H3BPG0_HUMAN Cytochrome *c* oxidase subunit 4 isoform 1, mitochondrial (Fragment) OS=Homo sapiens GN=COX4I1 PE=1 SV=1
sp|Q9UBX3|DIC_HUMAN	3.14	0.02829	6.89E-04	2.19E-04	Mitochondrial dicarboxylate carrier OS=Homo sapiens GN=SLC25A10 PE=1 SV=2
tr|G3V576|G3V576_HUMAN	3.13	0.01342	1.92E-03	6.15E-04	Heterogeneous nuclear ribonucleoproteins C1/C2 OS=Homo sapiens GN=HNRNPC PE=1 SV=1: tr|G3V575|G3V575_HUMAN Heterogeneous nuclear ribonucleoproteins C1/C2 (Fragment) OS=Homo sapiens GN=HNRNPC PE=1 SV=1: tr|G3V555|G3V555_HUMAN Heterogeneous nuclear ribonucleoproteins C1/C2 (Fragment) OS=Homo sapiens GN=HNRNPC PE=1 SV=1
sp|P07910|HNRPC_HUMAN	3.13	0.01342	1.45E-03	4.64E-04	Heterogeneous nuclear ribonucleoproteins C1/C2 OS=Homo sapiens GN=HNRNPC PE=1 SV=4
tr|B4DY08|B4DY08_HUMAN	3.13	0.01342	1.54E-03	4.93E-04	Heterogeneous nuclear ribonucleoproteins C1/C2 OS=Homo sapiens GN=HNRNPC PE=1 SV=1: tr|G3V4W0|G3V4W0_HUMAN Heterogeneous nuclear ribonucleoproteins C1/C2 (Fragment) OS=Homo sapiens GN=HNRNPC PE=1 SV=1: tr|G3V251|G3V251_HUMAN Heterogeneous nuclear ribonucleoproteins C1/C2 (Fragment) OS=Homo sapiens GN=HNRNPC PE=1 SV=1: tr|G3V3K6|G3V3K6_HUMAN Heterogeneous nuclear ribonucleoproteins C1/C2 (Fragment) OS=Homo sapiens GN=HNRNPC PE=1 SV=1: tr|G3V5X6|G3V5X6_HUMAN Heterogeneous nuclear ribonucleoproteins C1/C2 (Fragment) OS=Homo sapiens GN=HNRNPC PE=1 SV=1: tr|G3V4M8|G3V4M8_HUMAN Heterogeneous nuclear ribonucleoproteins C1/C2 (Fragment) OS=Homo sapiens GN=HNRNPC PE=1 SV=1: tr|G3V2H6|G3V2H6_HUMAN Heterogeneous nuclear ribonucleoproteins C1/C2 (Fragment) OS=Homo sapiens GN=HNRNPC PE=1 SV=1
tr|G3V2Q1|G3V2Q1_HUMAN	3.13	0.01342	1.46E-03	4.66E-04	Heterogeneous nuclear ribonucleoproteins C1/C2 OS=Homo sapiens GN=HNRNPC PE=1 SV=1
tr|G3V4C1|G3V4C1_HUMAN	3.13	0.01342	1.52E-03	4.87E-04	Heterogeneous nuclear ribonucleoproteins C1/C2 OS=Homo sapiens GN=HNRNPC PE=1 SV=1
sp|O15173|PGRC2_HUMAN	3.11	0.00180	7.94E-04	2.55E-04	Membrane-associated progesterone receptor component 2 OS=Homo sapiens GN=PGRMC2 PE=1 SV=1
sp|P05787|K2C8_HUMAN	3.02	0.01302	8.22E-03	2.72E-03	Keratin, type II cytoskeletal 8 OS=Homo sapiens GN=KRT8 PE=1 SV=7: tr|F8VUG2|F8VUG2_HUMAN Keratin, type II cytoskeletal 8 (Fragment) OS=Homo sapiens GN=KRT8 PE=1 SV=1: tr|F8VP67|F8VP67_HUMAN Keratin, type II cytoskeletal 8 (Fragment) OS=Homo sapiens GN=KRT8 PE=1 SV=1: tr|F8VRG4|F8VRG4_HUMAN Keratin, type II cytoskeletal 8 (Fragment) OS=Homo sapiens GN=KRT8 PE=1 SV=1
sp|P61353|RL27_HUMAN	2.98	0.03444	9.43E-04	3.17E-04	60S ribosomal protein L27 OS=Homo sapiens GN=RPL27 PE=1 SV=2: tr|K7EQQ9|K7EQQ9_HUMAN 60S ribosomal protein L27 OS=Homo sapiens GN=RPL27 PE=1 SV=1
tr|K7ELC7|K7ELC7_HUMAN	2.98	0.03444	8.91E-04	2.99E-04	60S ribosomal protein L27 (Fragment) OS=Homo sapiens GN=RPL27 PE=1 SV=1
tr|E9PCY7|E9PCY7_HUMAN	2.81	0.02643	1.33E-03	4.74E-04	Heterogeneous nuclear ribonucleoprotein H OS=Homo sapiens GN=HNRNPH1 PE=1 SV=1: tr|H0YBG7|H0YBG7_HUMAN Heterogeneous nuclear ribonucleoprotein H (Fragment) OS=Homo sapiens GN=HNRNPH1 PE=1 SV=1: tr|D6RBM0|D6RBM0_HUMAN Heterogeneous nuclear ribonucleoprotein H (Fragment) OS=Homo sapiens GN=HNRNPH1 PE=1 SV=1: tr|E5RGH4|E5RGH4_HUMAN Heterogeneous nuclear ribonucleoprotein H (Fragment) OS=Homo sapiens GN=HNRNPH1 PE=1 SV=1: tr|D6RIU0|D6RIU0_HUMAN Heterogeneous nuclear ribonucleoprotein H (Fragment) OS=Homo sapiens GN=HNRNPH1 PE=1 SV=1: tr|D6RFM3|D6RFM3_HUMAN Heterogeneous nuclear ribonucleoprotein H (Fragment) OS=Homo sapiens GN=HNRNPH1 PE=1 SV=1
tr|E7EMC6|E7EMC6_HUMAN	2.77	0.00863	5.78E-04	2.08E-04	Annexin OS=Homo sapiens GN=ANXA6 PE=1 SV=1
sp|P37108|SRP14_HUMAN	2.71	0.01021	8.50E-04	3.14E-04	Signal recognition particle 14 kDa protein OS=Homo sapiens GN=SRP14 PE=1 SV=2: tr|H0YLW0|H0YLW0_HUMAN Signal recognition particle 14 kDa protein OS=Homo sapiens GN=SRP14 PE=1 SV=1
sp|Q53GQ0|DHB12_HUMAN	2.42	0.01385	6.34E-04	2.62E-04	Estradiol 17-beta-dehydrogenase 12 OS=Homo sapiens GN=HSD17B12 PE=1 SV=2
sp|P04040|CATA_HUMAN	2.42	0.00728	7.06E-04	2.92E-04	Catalase OS=Homo sapiens GN=CAT PE=1 SV=3
tr|F8VVM2|F8VVM2_HUMAN	2.41	0.00909	8.74E-04	3.62E-04	Phosphate carrier protein, mitochondrial OS=Homo sapiens GN=SLC25A3 PE=1 SV=1
tr|H0YLA2|H0YLA2_HUMAN	2.30	0.00896	8.54E-04	3.71E-04	Signal recognition particle 14 kDa protein OS=Homo sapiens GN=SRP14 PE=1 SV=1
sp|P22087|FBRL_HUMAN	2.21	0.01012	8.83E-04	4.00E-04	rRNA 2′-O-methyltransferase fibrillarin OS=Homo sapiens GN=FBL PE=1 SV=2: tr|M0R299|M0R299_HUMAN rRNA 2′-O-methyltransferase fibrillarin (Fragment) OS=Homo sapiens GN=FBL PE=1 SV=1
tr|H3BNX8|H3BNX8_HUMAN	0.75	0.04600	1.27E-03	1.68E-04	Cytochrome c oxidase subunit 5A, mitochondrial OS=Homo sapiens GN=COX5A PE=1 SV=1
tr|H0Y449|H0Y449_HUMAN	−1.11	0.00212	2.27E-04	2.52E-03	Nuclease-sensitive element-binding protein 1 (Fragment) OS=Homo sapiens GN=YBX1 PE=1 SV=1
tr|H3BRN4|H3BRN4_HUMAN	−1.70	0.00099	4.66E-04	7.91E-04	4-aminobutyrate aminotransferase, mitochondrial OS=Homo sapiens GN=ABAT PE=1 SV=1: sp|P80404|GABT_HUMAN 4-aminobutyrate aminotransferase, mitochondrial OS=Homo sapiens GN=ABAT PE=1 SV=3
sp|P16401|H15_HUMAN	−1.74	0.04345	2.18E-04	3.79E-03	Histone H1.5 OS=Homo sapiens GN=HIST1H1B PE=1 SV=3
tr|H3BNQ7|H3BNQ7_HUMAN	−2.14	0.00576	3.69E-04	7.89E-04	4-aminobutyrate aminotransferase, mitochondrial OS=Homo sapiens GN=ABAT PE=1 SV=1
tr|A0A087WYT3|A0A087WYT3_HUMAN	−2.22	0.00267	4.43E-04	9.82E-04	Prostaglandin E synthase 3 OS=Homo sapiens GN=PTGES3 PE=4 SV=1
sp|Q15185|TEBP_HUMAN	−2.37	0.00333	4.54E-04	1.08E-03	Prostaglandin E synthase 3 OS=Homo sapiens GN=PTGES3 PE=1 SV=1
sp|O14979|HNRDL_HUMAN	−2.55	0.01357	1.96E-04	5.01E-04	Heterogeneous nuclear ribonucleoprotein D-like OS=Homo sapiens GN=HNRNPDL PE=1 SV=3
tr|A0A087WUK2|A0A087WUK2_HUMAN	−2.55	0.01357	2.27E-04	5.79E-04	Heterogeneous nuclear ribonucleoprotein D-like OS=Homo sapiens GN=HNRNPDL PE=4 SV=1
sp|P01009|A1AT_HUMAN	−2.74	0.02442	2.45E-04	6.73E-04	Alpha-1-antitrypsin OS=Homo sapiens GN=SERPINA1 PE=1 SV=3: tr|G3V2B9|G3V2B9_HUMAN Short peptide from AAT (Fragment) OS=Homo sapiens GN=SERPINA1 PE=1 SV=1: tr|G3V544|G3V544_HUMAN Short peptide from AAT (Fragment) OS=Homo sapiens GN=SERPINA1 PE=1 SV=1: tr|G3V387|G3V387_HUMAN Short peptide from AAT (Fragment) OS=Homo sapiens GN=SERPINA1 PE=1 SV=1: tr|G3V5R8|G3V5R8_HUMAN Short peptide from AAT (Fragment) OS=Homo sapiens GN=SERPINA1 PE=1 SV=1: tr|G3V4I7|G3V4I7_HUMAN Short peptide from AAT (Fragment) OS=Homo sapiens GN=SERPINA1 PE=4 SV=1
sp|P05455|LA_HUMAN	−2.88	0.02516	2.26E-04	6.52E-04	Lupus La protein OS=Homo sapiens GN=SSB PE=1 SV=2: tr|E7ERC4|E7ERC4_HUMAN Lupus La protein (Fragment) OS=Homo sapiens GN=SSB PE=1 SV=1: tr|E9PGX9|E9PGX9_HUMAN Lupus La protein (Fragment) OS=Homo sapiens GN=SSB PE=1 SV=1
sp|P10599|THIO_HUMAN	−3.01	0.04302	2.14E-04	6.44E-04	Thioredoxin OS=Homo sapiens GN=TXN PE=1 SV=3
tr|E7EMB3|E7EMB3_HUMAN	−3.13	0.04674	6.16E-04	1.93E-03	Calmodulin OS=Homo sapiens GN=CALM2 PE=1 SV=1
tr|H0Y7A7|H0Y7A7_HUMAN	−3.13	0.04674	6.45E-04	2.02E-03	Calmodulin (Fragment) OS=Homo sapiens GN=CALM2 PE=1 SV=1
tr|E7ETZ0|E7ETZ0_HUMAN	−3.13	0.04674	8.05E-04	2.52E-03	Calmodulin OS=Homo sapiens GN=CALM1 PE=1 SV=1
sp|P62158|CALM_HUMAN	−3.16	0.04206	8.10E-04	2.56E-03	Calmodulin OS=Homo sapiens GN=CALM1 PE=1 SV=2: tr|Q96HY3|Q96HY3_HUMAN CALM1 protein OS=Homo sapiens GN=CALM3 PE=1 SV=1: tr|G3V361|G3V361_HUMAN Calmodulin (Fragment) OS=Homo sapiens GN=CALM1 PE=1 SV=1
tr|J3QQX2|J3QQX2_HUMAN	−3.54	0.00702	2.84E-04	1.00E-03	Rho GDP-dissociation inhibitor 1 OS=Homo sapiens GN=ARHGDIA PE=1 SV=1: tr|J3KTF8|J3KTF8_HUMAN Rho GDP-dissociation inhibitor 1 (Fragment) OS=Homo sapiens GN=ARHGDIA PE=1 SV=3: tr|J3KS60|J3KS60_HUMAN Rho GDP-dissociation inhibitor 1 OS=Homo sapiens GN=ARHGDIA PE=1 SV=1
sp|P52565|GDIR1_HUMAN	−3.59	0.00515	3.27E-04	1.17E-03	Rho GDP-dissociation inhibitor 1 OS=Homo sapiens GN=ARHGDIA PE=1 SV=3
sp|P39687|AN32A_HUMAN	−3.84	0.01529	1.68E-04	6.45E-04	Acidic leucine-rich nuclear phosphoprotein 32 family member A OS=Homo sapiens GN=ANP32A PE=1 SV=1
sp|P25787|PSA2_HUMAN	−4.00	0.02378	3.39E-04	1.36E-03	Proteasome subunit alpha type-2 OS=Homo sapiens GN=PSMA2 PE=1 SV=2
sp|P17174|AATC_HUMAN	−4.13	0.01587	2.65E-04	1.09E-03	Aspartate aminotransferase, cytoplasmic OS=Homo sapiens GN=GOT1 PE=1 SV=3
tr|K7ER90|K7ER90_HUMAN	−4.13	0.03401	1.33E-04	5.49E-04	Eukaryotic translation initiation factor 3 subunit G (Fragment) OS=Homo sapiens GN=EIF3G PE=1 SV=1
sp|P06748|NPM_HUMAN	−4.20	0.02188	8.93E-04	3.75E-03	Nucleophosmin OS=Homo sapiens GN=NPM1 PE=1 SV=2: tr|E5RI98|E5RI98_HUMAN Nucleophosmin (Fragment) OS=Homo sapiens GN=NPM1 PE=1 SV=1
tr|J3QLC8|J3QLC8_HUMAN	−4.24	0.01244	1.73E-04	7.34E-04	60S ribosomal protein L17 OS=Homo sapiens GN=RPL17 PE=1 SV=1
sp|P49773|HINT1_HUMAN	−5.72	0.01114	6.04E-04	3.45E-03	Histidine triad nucleotide-binding protein 1 OS=Homo sapiens GN=HINT1 PE=1 SV=2
sp|Q86SX6|GLRX5_HUMAN	−6.14	0.04372	2.79E-04	1.71E-03	Glutaredoxin-related protein 5, mitochondrial OS=Homo sapiens GN=GLRX5 PE=1 SV=2
sp|Q15181|IPYR_HUMAN	−6.55	0.03416	8.78E-05	5.75E-04	Inorganic pyrophosphatase OS=Homo sapiens GN=PPA1 PE=1 SV=2
tr|H0YCY6|H0YCY6_HUMAN	−7.66	0.00032	7.86E-05	6.02E-04	FAD-AMP lyase (cyclizing) (Fragment) OS=Homo sapiens GN=DAK PE=1 SV=1

**Table 2:** List of 41 differentially abundant proteins for statistics between pcDNA3 x pcENS1.

**Locus**	**Fold Change**	***p*Value**	**Signal+(pcENS1)**	**Signal-(pcDNA3)**	**Description**
sp|Q9UK22|FBX2_HUMAN	2.887204251	0.00294	0.000565646	0.000195915	F-box only protein 2 OS=Homo sapiens GN=FBXO2 PE=1 SV=2
tr|F2Z2V0|F2Z2V0_HUMAN	2.448891458	0.01885	0.000415618	0.000169717	Copine-1 (Fragment) OS=Homo sapiens GN=CPNE1 PE=1 SV=1
sp|P05386|RLA1_HUMAN	2.20861757	0.01819	0.002338141	0.001058645	60S acidic ribosomal protein P1 OS=Homo sapiens GN=RPLP1 PE=1 SV=1
sp|P43243|MATR3_HUMAN	2.106781667	0.00112	0.000626087	0.000297177	Matrin-3 OS=Homo sapiens GN=MATR3 PE=1 SV=2: tr|B3KM87|B3KM87_HUMAN Matrin-3 OS=Homo sapiens GN=MATR3 PE=1 SV=1
tr|A8MXP9|A8MXP9_HUMAN	2.106781667	0.00112	0.000592509	0.000281239	Matrin-3 OS=Homo sapiens GN=MATR3 PE=1 SV=1
sp|P51149|RAB7A_HUMAN	1.938939554	0.00429	0.000768084	0.000396136	Ras-related protein Rab-7a OS=Homo sapiens GN=RAB7A PE=1 SV=1: tr|C9J592|C9J592_HUMAN Ras-related protein Rab-7a (Fragment) OS=Homo sapiens GN=RAB7A PE=1 SV=1
sp|Q9P035|HACD3_HUMAN	1.842287267	0.00587	0.00044159	0.000239697	Very-long-chain (3R)-3-hydroxyacyl-CoA dehydratase 3 OS=Homo sapiens GN=PTPLAD1 PE=1 SV=2
sp|P52926|HMGA2_HUMAN	−0.002597139	0.02916	0.000932861	0.002422769	High mobility group protein HMGI-C OS=Homo sapiens GN=HMGA2 PE=1 SV=1
tr|F5H2A4|F5H2A4_HUMAN	−0.002597139	0.02916	0.00086171	0.002237981	High mobility group protein HMGI-C OS=Homo sapiens GN=HMGA2 PE=1 SV=1
tr|F5H6H0|F5H6H0_HUMAN	−0.002597139	0.02916	0.000691713	0.001796475	High mobility group protein HMGI-C OS=Homo sapiens GN=HMGA2 PE=1 SV=1
tr|J3QRW1|J3QRW1_HUMAN	−0.018702242	0.00638	0.000248201	0.000464192	26S protease regulatory subunit 8 (Fragment) OS=Homo sapiens GN=PSMC5 PE=1 SV=1
sp|P00338|LDHA_HUMAN	−0.267759962	0.03438	0.000722573	0.001934761	>L-lactate dehydrogenase A chain OS=Homo sapiens GN=LDHA PE=1 SV=2: tr|F5GXY2|F5GXY2_HUMAN l-lactate dehydrogenase A chain (Fragment) OS=Homo sapiens GN=LDHA PE=1 SV=3
sp|P02765|FETUA_HUMAN	−0.475079452	0.0123	0.000111731	0.00053081	Alpha-2-HS-glycoprotein OS=Homo sapiens GN=AHSG PE=1 SV=1
sp|P08238|HS90B_HUMAN	−1.523033786	0.0036	0.002236377	0.003406078	Heat shock protein HSP 90-beta OS=Homo sapiens GN=HSP90AB1 PE=1 SV=4
sp|P02545|LMNA_HUMAN	−1.560530275	0.00312	0.000879213	0.001372039	Prelamin-A/C OS=Homo sapiens GN=LMNA PE=1 SV=1
sp|P39023|RL3_HUMAN	−2.033045505	0.00327	0.000226732	0.000460956	60S ribosomal protein L3 OS=Homo sapiens GN=RPL3 PE=1 SV=2: tr|G5E9G0|G5E9G0_HUMAN 60S ribosomal protein L3 OS=Homo sapiens GN=RPL3 PE=1 SV=1: tr|B5MCW2|B5MCW2_HUMAN 60S ribosomal protein L3 (Fragment) OS=Homo sapiens GN=RPL3 PE=1 SV=1
sp|P02768|ALBU_HUMAN	−2.302973671	0.00479	0.000275409	0.000634261	Serum albumin OS=Homo sapiens GN=ALB PE=1 SV=2
sp|P26373|RL13_HUMAN	−2.352456304	0.0203	0.000324859	0.000764216	60S ribosomal protein L13 OS=Homo sapiens GN=RPL13 PE=1 SV=4: tr|H3BUK8|H3BUK8_HUMAN 60S ribosomal protein L13 (Fragment) OS=Homo sapiens GN=RPL13 PE=1 SV=1: tr|J3QSB4|J3QSB4_HUMAN 60S ribosomal protein L13 (Fragment) OS=Homo sapiens GN=RPL13 PE=1 SV=1
tr|A0A087X1S2|A0A087X1S2_HUMAN	−2.401479196	0.00478	0.000370535	0.000889831	Nuclease-sensitive element-binding protein 1 OS=Homo sapiens GN=YBX1 PE=4 SV=1
tr|A0A087WZH7|A0A087WZH7_HUMAN	−2.408424932	0.00411	0.000303789	0.000731652	Myristoylated alanine-rich C-kinase substrate OS=Homo sapiens GN=MARCKS PE=4 SV=1
tr|A0A087WWU8|A0A087WWU8_HUMAN	−2.440187743	0.00449	0.000253765	0.000619234	Tropomyosin alpha-3 chain OS=Homo sapiens GN=TPM3 PE=4 SV=1
sp|P29966|MARCS_HUMAN	−2.684863748	0.00191	0.00026381	0.000708295	Myristoylated alanine-rich C-kinase substrate OS=Homo sapiens GN=MARCKS PE=1 SV=4
sp|Q9GZT3|SLIRP_HUMAN	−2.730197953	0.00514	0.000172275	0.000470346	SRA stem-loop-interacting RNA-binding protein, mitochondrial OS=Homo sapiens GN=SLIRP PE=1 SV=1
tr|G3V2S9|G3V2S9_HUMAN	−2.730197953	0.00514	0.000151436	0.000413449	SRA stem-loop-interacting RNA-binding protein, mitochondrial OS=Homo sapiens GN=SLIRP PE=1 SV=1
tr|H0YJ40|H0YJ40_HUMAN	−2.730197953	0.00514	0.000195604	0.000534038	SRA stem-loop-interacting RNA-binding protein, mitochondrial (Fragment) OS=Homo sapiens GN=SLIRP PE=1 SV=1
tr|A0A087WUN7|A0A087WUN7_HUMAN	−2.730197953	0.00514	0.000204109	0.000557257	SRA stem-loop-interacting RNA-binding protein, mitochondrial OS=Homo sapiens GN=SLIRP PE=4 SV=1
tr|G3V4X6|G3V4X6_HUMAN	−2.730197953	0.00514	0.000191612	0.00052314	SRA stem-loop-interacting RNA-binding protein, mitochondrial OS=Homo sapiens GN=SLIRP PE=1 SV=1
sp|P53004|BIEA_HUMAN	−2.899141374	0.03897	0.000143578	0.000416253	Biliverdin reductase A OS=Homo sapiens GN=BLVRA PE=1 SV=2: tr|C9J1E1|C9J1E1_HUMAN Biliverdin reductase A (Fragment) OS=Homo sapiens GN=BLVRA PE=1 SV=1
tr|H3BT36|H3BT36_HUMAN	−3.082880329	0.02293	0.000536524	0.001654038	Proteasome subunit alpha type-2 OS=Homo sapiens GN=PSMA2 PE=4 SV=1
sp|P62269|RS18_HUMAN	−3.304625932	0.02144	0.000280286	0.000926242	40S ribosomal protein S18 OS=Homo sapiens GN=RPS18 PE=1 SV=3: tr|J3JS69|J3JS69_HUMAN 40S ribosomal protein S18 OS=Homo sapiens GN=RPS18 PE=1 SV=1
tr|B7WNR0|B7WNR0_HUMAN	−3.482472238	0.0473	0.000142247	0.00049537	Serum albumin OS=Homo sapiens GN=ALB PE=1 SV=1
tr|D6RHD5|D6RHD5_HUMAN	−3.482472238	0.0473	0.000153093	0.000533143	Serum albumin OS=Homo sapiens GN=ALB PE=1 SV=1
tr|H0YA55|H0YA55_HUMAN	−3.482472238	0.0473	0.000154779	0.000539015	Serum albumin (Fragment) OS=Homo sapiens GN=ALB PE=1 SV=1
tr|H7C367|H7C367_HUMAN	−3.818005689	0.03869	0.000206727	0.000789284	Non-POU domain-containing octamer-binding protein (Fragment) OS=Homo sapiens GN=NONO PE=1 SV=3
tr|A0A087X0X3|A0A087X0X3_HUMAN	−3.895589738	0.04525	0.000131893	0.000513803	Heterogeneous nuclear ribonucleoprotein M OS=Homo sapiens GN=HNRNPM PE=4 SV=1
sp|Q5VTE0|EF1A3_HUMAN	−4.17671178	0.00362	0.001520654	0.006351333	Putative elongation factor 1-alpha-like 3 OS=Homo sapiens GN=EEF1A1P5 PE=5 SV=1
tr|C9J0J7|C9J0J7_HUMAN	−4.228640752	0.01546	0.000141501	0.000598356	Profilin-2 OS=Homo sapiens GN=PFN2 PE=1 SV=1
tr|C9JQ45|C9JQ45_HUMAN	−4.228640752	0.01546	0.00011706	0.000495003	Profilin OS=Homo sapiens GN=PFN2 PE=1 SV=1
tr|Q5TA01|Q5TA01_HUMAN	−4.374529519	0.01927	0.000236106	0.001032853	Glutathione S-transferase omega-1 (Fragment) OS=Homo sapiens GN=GSTO1 PE=1 SV=1
sp|Q9H9B4|SFXN1_HUMAN	−4.423436439	0.01596	0.000148649	0.000657539	Sideroflexin-1 OS=Homo sapiens GN=SFXN1 PE=1 SV=4: tr|D6RFI0|D6RFI0_HUMAN Sideroflexin-1 (Fragment) OS=Homo sapiens GN=SFXN1 PE=1 SV=3
sp|P25786|PSA1_HUMAN	−9.640254654	0.00328	4.97959E-05	0.000480045	Proteasome subunit alpha type-1 OS=Homo sapiens GN=PSMA1 PE=1 SV=1: tr|F5GX11|F5GX11_HUMAN Proteasome subunit alpha type-1 OS=Homo sapiens GN=PSMA1 PE=1 SV=1

**Table 3:** List of 81 differentially abundant proteins for statistics between pMAXGFP x pcENS1.

**Locus**	**Fold Change**	***p*Value**	**Signal+(pcENS1)**	**Signal-(pMAXGFP)**	**Description**
tr|E9PIZ4|E9PIZ4_HUMAN	4.27	0.02884	5.1564100956E-04	1.2067638747E-04	Cysteine and histidine-rich domain-containing protein 1 OS=Homo sapiens GN=CHORDC1 PE=1 SV=1
tr|H0YMI6|H0YMI6_HUMAN	2.63	0.04622	3.6248916762E-04	1.3785223116E-04	Proteasome subunit alpha type (Fragment) OS=Homo sapiens GN=PSMA4 PE=1 SV=1
sp|P60900|PSA6_HUMAN	2.51	0.01497	6.5820719273E-04	2.6200153475E-04	Proteasome subunit alpha type-6 OS=Homo sapiens GN=PSMA6 PE=1 SV=1
tr|G3V295|G3V295_HUMAN	2.51	0.01497	7.9763039119E-04	3.1749939680E-04	Proteasome subunit alpha type OS=Homo sapiens GN=PSMA6 PE=1 SV=1
tr|G3V3I1|G3V3I1_HUMAN	2.51	0.01497	1.0940470906E-03	4.3548903750E-04	Proteasome subunit alpha type OS=Homo sapiens GN=PSMA6 PE=1 SV=1
tr|G3V3U4|G3V3U4_HUMAN	2.51	0.01497	1.5132613964E-03	6.0235866869E-04	Proteasome subunit alpha type OS=Homo sapiens GN=PSMA6 PE=1 SV=1
tr|G3V5Z7|G3V5Z7_HUMAN	2.51	0.01497	6.4253559290E-04	2.5576340297E-04	Proteasome subunit alpha type OS=Homo sapiens GN=PSMA6 PE=1 SV=1
sp|P61626|LYSC_HUMAN	2.46	0.00250	8.0788085952E-04	3.2813609299E-04	Lysozyme C OS=Homo sapiens GN=LYZ PE=1 SV=1
sp|P62081|RS7_HUMAN	2.07	0.00203	4.7837342096E-04	2.3069960621E-04	40S ribosomal protein S7 OS=Homo sapiens GN=RPS7 PE=1 SV=1
sp|O43175|SERA_HUMAN	2.05	0.02052	3.5415580623E-04	1.7288143062E-04	>D-3-phosphoglycerate dehydrogenase OS=Homo sapiens GN=PHGDH PE=1 SV=4
tr|M0R210|M0R210_HUMAN	1.88	0.03982	1.8803502747E-03	1.0008461759E-03	40S ribosomal protein S16 OS=Homo sapiens GN=RPS16 PE=1 SV=1
sp|P62249|RS16_HUMAN	1.84	0.03469	1.6295554871E-03	8.8430929241E-04	40S ribosomal protein S16 OS=Homo sapiens GN=RPS16 PE=1 SV=2
tr|A0A087WZ27|A0A087WZ27_HUMAN	1.84	0.03469	1.6295554871E-03	8.8430929241E-04	Zinc finger protein 90 OS=Homo sapiens GN=ZNF90 PE=4 SV=1
sp|P46782|RS5_HUMAN	1.82	0.04115	1.3188765458E-03	7.2344215749E-04	40S ribosomal protein S5 OS=Homo sapiens GN=RPS5 PE=1 SV=4: tr|M0R0F0|M0R0F0_HUMAN 40S ribosomal protein S5 (Fragment) OS=Homo sapiens GN=RPS5 PE=1 SV=1
sp|P50395|GDIB_HUMAN	1.82	0.04377	7.3807276416E-04	4.0635192954E-04	Rab GDP dissociation inhibitor beta OS=Homo sapiens GN=GDI2 PE=1 SV=2: tr|V9GYF8|V9GYF8_HUMAN Rab GDP dissociation inhibitor beta (Fragment) OS=Homo sapiens GN=GDI2 PE=1 SV=1
sp|P62136|PP1A_HUMAN	1.81	0.04937	7.1055348515E-04	3.9154015764E-04	Serine/threonine-protein phosphatase PP1-alpha catalytic subunit OS=Homo sapiens GN=PPP1CA PE=1 SV=1
tr|K7ERG4|K7ERG4_HUMAN	1.79	0.03148	9.9775810350E-04	5.5628189049E-04	Small nuclear ribonucleoprotein Sm D2 OS=Homo sapiens GN=SNRPD2 PE=1 SV=1
tr|M0R0R2|M0R0R2_HUMAN	1.79	0.04224	1.1751143601E-03	6.5592088945E-04	40S ribosomal protein S5 OS=Homo sapiens GN=RPS5 PE=1 SV=1
sp|P47755|CAZA2_HUMAN	1.76	0.00907	3.8503391537E-04	2.1892360270E-04	F-actin-capping protein subunit alpha-2 OS=Homo sapiens GN=CAPZA2 PE=1 SV=3
tr|A8MXQ1|A8MXQ1_HUMAN	1.58	0.00171	1.8271019599E-03	1.1545048441E-03	Pituitary tumor-transforming gene 1 protein-interacting protein OS=Homo sapiens GN=PTTG1IP PE=4 SV=1
sp|P43243|MATR3_HUMAN	1.56	0.00335	6.2608664580E-04	4.0083578369E-04	Matrin-3 OS=Homo sapiens GN=MATR3 PE=1 SV=2: tr|B3KM87|B3KM87_HUMAN Matrin-3 OS=Homo sapiens GN=MATR3 PE=1 SV=1
tr|A8MXP9|A8MXP9_HUMAN	1.56	0.00335	5.9250881452E-04	3.7933844557E-04	Matrin-3 OS=Homo sapiens GN=MATR3 PE=1 SV=1
tr|E9PSD5|E9PSD5_HUMAN	0.43	0.02884	4.9845297591E-04	1.1665384122E-04	Cysteine and histidine-rich domain-containing protein 1 OS=Homo sapiens GN=CHORDC1 PE=1 SV=1
sp|P18085|ARF4_HUMAN	0.18	0.01873	1.0283317157E-03	5.6312821438E-04	ADP-ribosylation factor 4 OS=Homo sapiens GN=ARF4 PE=1 SV=3: tr|C9JPM4|C9JPM4_HUMAN ADP-ribosylation factor 4 (Fragment) OS=Homo sapiens GN=ARF4 PE=1 SV=1
tr|A0A087X1Z3|A0A087X1Z3_HUMAN	−0.20	0.00919	2.9662475890E-04	5.9412009618E-04	Proteasome activator complex subunit 2 OS=Homo sapiens GN=PSME2 PE=4 SV=1
tr|H0YM70|H0YM70_HUMAN	−0.20	0.00919	3.3045038930E-04	6.6187063346E-04	Proteasome activator complex subunit 2 OS=Homo sapiens GN=PSME2 PE=1 SV=1: tr|H0YKU2|H0YKU2_HUMAN Proteasome activator complex subunit 2 (Fragment) OS=Homo sapiens GN=PSME2 PE=1 SV=1
tr|H3BT71|H3BT71_HUMAN	−0.22	0.03725	3.6164761545E-04	7.8828832196E-04	RNA-binding motif protein, X chromosome, N-terminally processed OS=Homo sapiens GN=RBMX PE=1 SV=1
tr|H0YMF4|H0YMF4_HUMAN	−0.24	0.01281	4.5610249693E-04	1.0891334963E-03	60S ribosomal protein L28 OS=Homo sapiens GN=RPL28 PE=1 SV=1
sp|Q92928|RAB1C_HUMAN	−0.33	0.00379	1.7941339393E-04	5.9502701116E-04	Putative Ras-related protein Rab-1C OS=Homo sapiens GN=RAB1C PE=5 SV=2
sp|Q9H0U4|RAB1B_HUMAN	−0.33	0.00379	1.7941339393E-04	5.9502701116E-04	Ras-related protein Rab-1B OS=Homo sapiens GN=RAB1B PE=1 SV=1
sp|P08238|HS90B_HUMAN	−1.19	0.00037	2.2363769567E-03	2.6541184171E-03	Heat shock protein HSP 90-beta OS=Homo sapiens GN=HSP90AB1 PE=1 SV=4
sp|P15531|NDKA_HUMAN	−1.38	0.00005	1.9216269100E-03	2.6508648328E-03	Nucleoside diphosphate kinase A OS=Homo sapiens GN=NME1 PE=1 SV=1
sp|P22570|ADRO_HUMAN	−1.40	0.00127	4.9680105400E-04	6.9510914113E-04	NADPH:adrenodoxin oxidoreductase, mitochondrial OS=Homo sapiens GN=FDXR PE=1 SV=3
sp|P02545|LMNA_HUMAN	−1.41	0.00262	8.7921299898E-04	1.2356451265E-03	Prelamin-A/C OS=Homo sapiens GN=LMNA PE=1 SV=1
sp|Q8NBS9|TXND5_HUMAN	−1.55	0.01047	4.3710044382E-04	6.7551841613E-04	Thioredoxin domain-containing protein 5 OS=Homo sapiens GN=TXNDC5 PE=1 SV=2
tr|K7ES89|K7ES89_HUMAN	−1.57	0.00284	3.3391802783E-04	5.2383721696E-04	Dual-specificity protein phosphatase 3 (Fragment) OS=Homo sapiens GN=DUSP3 PE=1 SV=1
sp|P60953|CDC42_HUMAN	−1.59	0.01599	5.7952351298E-04	9.2218098533E-04	Cell division control protein 42 homolog OS=Homo sapiens GN=CDC42 PE=1 SV=2: tr|Q5JYX0|Q5JYX0_HUMAN Cell division control protein 42 homolog (Fragment) OS=Homo sapiens GN=CDC42 PE=1 SV=1
tr|E3W990|E3W990_HUMAN	−1.74	0.00295	2.0678393968E-04	3.6029249723E-04	Sequestosome-1 (Fragment) OS=Homo sapiens GN=SQSTM1 PE=1 SV=1
sp|Q14847|LASP1_HUMAN	−1.79	0.01080	5.3129711808E-04	9.5247256550E-04	LIM and SH3 domain protein 1 OS=Homo sapiens GN=LASP1 PE=1 SV=2: tr|C9J9W2|C9J9W2_HUMAN LIM and SH3 domain protein 1 (Fragment) OS=Homo sapiens GN=LASP1 PE=1 SV=1
tr|G3V1V0|G3V1V0_HUMAN	−1.80	0.01301	4.6887694121E-04	8.4429729734E-04	Myosin, light polypeptide 6, alkali, smooth muscle and non-muscle, isoform CRA_c OS=Homo sapiens GN=PDE6H PE=4 SV=1: tr|F8W1R7|F8W1R7_HUMAN Retinal cone rhodopsin-sensitive cGMP 3׳,5׳-cyclic phosphodiesterase subunit gamma OS=Homo sapiens GN=PDE6H PE=4 SV=1
sp|P60660|MYL6_HUMAN	−1.80	0.01301	4.9992839427E-04	9.0021102564E-04	Myosin light polypeptide 6 OS=Homo sapiens GN=MYL6 PE=1 SV=2
tr|B7Z6Z4|B7Z6Z4_HUMAN	−1.80	0.01301	3.1718146023E-04	5.7114228937E-04	Retinal cone rhodopsin-sensitive cGMP 3׳,5׳-cyclic phosphodiesterase subunit gamma OS=Homo sapiens GN=PDE6H PE=2 SV=1
tr|F8VPF3|F8VPF3_HUMAN	−1.80	0.01301	5.8068605796E-04	1.0456297298E-03	Retinal cone rhodopsin-sensitive cGMP 3׳,5׳-cyclic phosphodiesterase subunit gamma (Fragment) OS=Homo sapiens GN=PDE6H PE=4 SV=1
tr|G8JLA2|G8JLA2_HUMAN	−1.80	0.01301	4.9663939168E-04	8.9428858468E-04	Retinal cone rhodopsin-sensitive cGMP 3׳,5׳-cyclic phosphodiesterase subunit gamma OS=Homo sapiens GN=PDE6H PE=4 SV=1
tr|J3KND3|J3KND3_HUMAN	−1.80	0.01301	4.9663939168E-04	8.9428858468E-04	Retinal cone rhodopsin-sensitive cGMP 3׳,5׳-cyclic phosphodiesterase subunit gamma OS=Homo sapiens GN=PDE6H PE=4 SV=1
sp|P00338|LDHA_HUMAN	−1.91	0.04007	7.2257279358E-04	1.3809825096E-03	>L-lactate dehydrogenase A chain OS=Homo sapiens GN=LDHA PE=1 SV=2: tr|F5GXY2|F5GXY2_HUMAN l-lactate dehydrogenase A chain (Fragment) OS=Homo sapiens GN=LDHA PE=1 SV=3
sp|O95292|VAPB_HUMAN	−1.92	0.02424	2.7412268027E-04	5.2623836772E-04	Vesicle-associated membrane protein-associated protein B/C OS=Homo sapiens GN=VAPB PE=1 SV=3
tr|H7C2I1|H7C2I1_HUMAN	−1.99	0.01036	2.1368459062E-04	4.2559598943E-04	Protein arginine N-methyltransferase 1 OS=Homo sapiens GN=PRMT1 PE=1 SV=1: sp|Q99873|ANM1_HUMAN Protein arginine N-methyltransferase 1 OS=Homo sapiens GN=PRMT1 PE=1 SV=2: tr|E9PKG1|E9PKG1_HUMAN Protein arginine N-methyltransferase 1 OS=Homo sapiens GN=PRMT1 PE=1 SV=1
sp|Q9UL46|PSME2_HUMAN	−2.00	0.00919	3.1524137556E-04	6.3140796832E-04	Proteasome activator complex subunit 2 OS=Homo sapiens GN=PSME2 PE=1 SV=4
tr|A0A087WZH7|A0A087WZH7_HUMAN	−2.05	0.01540	3.0378860599E-04	6.2309294752E-04	Myristoylated alanine-rich C-kinase substrate OS=Homo sapiens GN=MARCKS PE=4 SV=1
sp|Q9NX63|MIC19_HUMAN	−2.06	0.04956	2.1744009773E-04	4.4818711141E-04	MICOS complex subunit MIC19 OS=Homo sapiens GN=CHCHD3 PE=1 SV=1
tr|C9JRZ6|C9JRZ6_HUMAN	−2.06	0.04956	2.1275388873E-04	4.3852790643E-04	MICOS complex subunit MIC19 OS=Homo sapiens GN=CHCHD3 PE=1 SV=1
sp|Q92688|AN32B_HUMAN	−2.16	0.04445	2.5741410440E-04	5.5640940806E-04	Acidic leucine-rich nuclear phosphoprotein 32 family member B OS=Homo sapiens GN=ANP32B PE=1 SV=1: tr|Q5T6W8|Q5T6W8_HUMAN Acidic leucine-rich nuclear phosphoprotein 32 family member B (Fragment) OS=Homo sapiens GN=ANP32B PE=1 SV=1
tr|H0Y6E7|H0Y6E7_HUMAN	−2.18	0.03725	3.6660169237E-04	7.9908679213E-04	RNA-binding motif protein, X chromosome, N-terminally processed (Fragment) OS=Homo sapiens GN=RBMX PE=1 SV=2
tr|C9J4S4|C9J4S4_HUMAN	−2.19	0.00627	4.4976713953E-04	9.8605217655E-04	Ras-related protein Rab-7a OS=Homo sapiens GN=RAB7A PE=1 SV=1
sp|O15143|ARC1B_HUMAN	−2.20	0.00773	1.6888176270E-04	3.7175544430E-04	Actin-related protein 2/3 complex subunit 1B OS=Homo sapiens GN=ARPC1B PE=1 SV=3: tr|C9JFG9|C9JFG9_HUMAN Actin-related protein 2/3 complex subunit 1B (Fragment) OS=Homo sapiens GN=ARPC1B PE=1 SV=3: tr|C9J6C8|C9J6C8_HUMAN Actin-related protein 2/3 complex subunit 1B (Fragment) OS=Homo sapiens GN=ARPC1B PE=1 SV=3: tr|C9JQM8|C9JQM8_HUMAN Actin-related protein 2/3 complex subunit 1B (Fragment) OS=Homo sapiens GN=ARPC1B PE=1 SV=3: tr|C9JEY1|C9JEY1_HUMAN Actin-related protein 2/3 complex subunit 1B (Fragment) OS=Homo sapiens GN=ARPC1B PE=1 SV=1: tr|F8VXW2|F8VXW2_HUMAN Actin-related protein 2/3 complex subunit 1B OS=Homo sapiens GN=ARPC1B PE=1 SV=2: tr|C9J4Z7|C9J4Z7_HUMAN Actin-related protein 2/3 complex subunit 1B (Fragment) OS=Homo sapiens GN=ARPC1B PE=1 SV=1: tr|C9K057|C9K057_HUMAN Actin-related protein 2/3 complex subunit 1B (Fragment) OS=Homo sapiens GN=ARPC1B PE=1 SV=1: tr|C9JBJ7|C9JBJ7_HUMAN Actin-related protein 2/3 complex subunit 1B (Fragment) OS=Homo sapiens GN=ARPC1B PE=1 SV=1: tr|C9JTT6|C9JTT6_HUMAN Actin-related protein 2/3 complex subunit 1B (Fragment) OS=Homo sapiens GN=ARPC1B PE=1 SV=3
tr|K7ELC7|K7ELC7_HUMAN	−2.22	0.03550	3.7971620398E-04	8.4465213197E-04	60S ribosomal protein L27 (Fragment) OS=Homo sapiens GN=RPL27 PE=1 SV=1
sp|P29966|MARCS_HUMAN	−2.30	0.00963	2.6381044628E-04	6.0621816617E-04	Myristoylated alanine-rich C-kinase substrate OS=Homo sapiens GN=MARCKS PE=1 SV=4
tr|H3BT36|H3BT36_HUMAN	−2.45	0.00347	5.3652366733E-04	1.3139040782E-03	Proteasome subunit alpha type-2 OS=Homo sapiens GN=PSMA2 PE=4 SV=1
sp|P46779|RL28_HUMAN	−2.59	0.00655	3.4415463721E-04	8.9038650796E-04	60S ribosomal protein L28 OS=Homo sapiens GN=RPL28 PE=1 SV=3
tr|H0YKD8|H0YKD8_HUMAN	−2.59	0.00655	2.7734814881E-04	7.1754677406E-04	60S ribosomal protein L28 OS=Homo sapiens GN=RPL28 PE=1 SV=1
tr|H0YLP6|H0YLP6_HUMAN	−2.59	0.00655	5.2976612693E-04	1.3705949617E-03	60S ribosomal protein L28 OS=Homo sapiens GN=RPL28 PE=1 SV=1
sp|O00483|NDUA4_HUMAN	−2.74	0.03035	3.7754188433E-04	1.0331761575E-03	Cytochrome c oxidase subunit NDUFA4 OS=Homo sapiens GN=NDUFA4 PE=1 SV=1
sp|Q15274|NADC_HUMAN	−2.75	0.00691	2.0572201432E-04	5.6661625682E-04	Nicotinate-nucleotide pyrophosphorylase [carboxylating] OS=Homo sapiens GN=QPRT PE=1 SV=3: tr|C9JCJ5|C9JCJ5_HUMAN Uncharacterized protein (Fragment) OS=Homo sapiens PE=4 SV=5
tr|H7BZ11|H7BZ11_HUMAN	−3.07	0.00710	2.2555444718E-04	6.9288128636E-04	Protein RPL36A-HNRNPH2 OS=Homo sapiens GN=RPL36A-HNRNPH2 PE=3 SV=2
tr|J3KQN4|J3KQN4_HUMAN	−3.07	0.00710	1.8743256878E-04	5.7577459007E-04	60S ribosomal protein L36a OS=Homo sapiens GN=RPL36A PE=3 SV=1: sp|P83881|RL36A_HUMAN 60S ribosomal protein L36a OS=Homo sapiens GN=RPL36A PE=1 SV=2: tr|R4GN19|R4GN19_HUMAN 60S ribosomal protein L36a OS=Homo sapiens GN=RPL36A PE=4 SV=1
sp|Q969Q0|RL36L_HUMAN	−3.07	0.00710	2.5108891290E-04	7.7132067726E-04	60S ribosomal protein L36a-like OS=Homo sapiens GN=RPL36AL PE=1 SV=3
tr|H0Y5B4|H0Y5B4_HUMAN	−3.07	0.00710	2.3763772114E-04	7.2999992670E-04	60S ribosomal protein L36a OS=Homo sapiens GN=RPL36A PE=3 SV=2
tr|D6RHD5|D6RHD5_HUMAN	−3.10	0.01904	1.5309327240E-04	4.7403637068E-04	Serum albumin OS=Homo sapiens GN=ALB PE=1 SV=1
tr|B7WNR0|B7WNR0_HUMAN	−3.10	0.01904	1.4224658306E-04	4.4045079786E-04	Serum albumin OS=Homo sapiens GN=ALB PE=1 SV=1
tr|H0YA55|H0YA55_HUMAN	−3.10	0.01904	1.5477932166E-04	4.7925703555E-04	Serum albumin (Fragment) OS=Homo sapiens GN=ALB PE=1 SV=1
sp|Q9HAV7|GRPE1_HUMAN	−3.24	0.02180	1.2662540785E-04	4.1001496166E-04	GrpE protein homolog 1, mitochondrial OS=Homo sapiens GN=GRPEL1 PE=1 SV=2
sp|P62269|RS18_HUMAN	−3.24	0.01564	2.8028634126E-04	9.0884462029E-04	40S ribosomal protein S18 OS=Homo sapiens GN=RPS18 PE=1 SV=3: tr|J3JS69|J3JS69_HUMAN 40S ribosomal protein S18 OS=Homo sapiens GN=RPS18 PE=1 SV=1
tr|C9JQB3|C9JQB3_HUMAN	−3.26	0.01641	1.1866193463E-04	3.8718514857E-04	Ras-related protein Ral-B (Fragment) OS=Homo sapiens GN=RALB PE=1 SV=1
tr|E9PLD0|E9PLD0_HUMAN	−3.70	0.04512	1.5238542031E-04	5.6381042429E-04	Ras-related protein Rab-1B OS=Homo sapiens GN=RAB1B PE=3 SV=1
sp|Q9UN86|G3BP2_HUMAN	−3.81	0.00228	1.1344218542E-04	4.3192164871E-04	Ras GTPase-activating protein-binding protein 2 OS=Homo sapiens GN=G3BP2 PE=1 SV=2: tr|D6RB17|D6RB17_HUMAN Ras GTPase-activating protein-binding protein 2 (Fragment) OS=Homo sapiens GN=G3BP2 PE=1 SV=1: tr|D6RAC7|D6RAC7_HUMAN Ras GTPase-activating protein-binding protein 2 (Fragment) OS=Homo sapiens GN=G3BP2 PE=1 SV=1: tr|D6RGJ4|D6RGJ4_HUMAN Ras GTPase-activating protein-binding protein 2 (Fragment) OS=Homo sapiens GN=G3BP2 PE=1 SV=1: tr|D6RBW8|D6RBW8_HUMAN Ras GTPase-activating protein-binding protein 2 (Fragment) OS=Homo sapiens GN=G3BP2 PE=1 SV=3: tr|D6RE13|D6RE13_HUMAN Ras GTPase-activating protein-binding protein 2 (Fragment) OS=Homo sapiens GN=G3BP2 PE=1 SV=3: tr|D6RBR0|D6RBR0_HUMAN Ras GTPase-activating protein-binding protein 2 (Fragment) OS=Homo sapiens GN=G3BP2 PE=1 SV=1: tr|D6REX8|D6REX8_HUMAN Ras GTPase-activating protein-binding protein 2 (Fragment) OS=Homo sapiens GN=G3BP2 PE=1 SV=1: tr|D6RBM9|D6RBM9_HUMAN Ras GTPase-activating protein-binding protein 2 (Fragment) OS=Homo sapiens GN=G3BP2 PE=1 SV=3: tr|D6R9A4|D6R9A4_HUMAN Ras GTPase-activating protein-binding protein 2 (Fragment) OS=Homo sapiens GN=G3BP2 PE=1 SV=1: tr|D6R9X5|D6R9X5_HUMAN Ras GTPase-activating protein-binding protein 2 (Fragment) OS=Homo sapiens GN=G3BP2 PE=1 SV=1
tr|M0QXU7|M0QXU7_HUMAN	−3.82	0.03844	9.5943708277E-05	3.6629063166E-04	Mitochondrial import inner membrane translocase subunit TIM44 (Fragment) OS=Homo sapiens GN=TIMM44 PE=1 SV=1
sp|Q9H9B4|SFXN1_HUMAN	−4.04	0.00033	1.4864899148E-04	6.0096890241E-04	Sideroflexin-1 OS=Homo sapiens GN=SFXN1 PE=1 SV=4: tr|D6RFI0|D6RFI0_HUMAN Sideroflexin-1 (Fragment) OS=Homo sapiens GN=SFXN1 PE=1 SV=3
tr|J3KRE2|J3KRE2_HUMAN	−4.63	0.02210	1.0372838583E-04	4.8012684663E-04	Rho GDP-dissociation inhibitor 1 OS=Homo sapiens GN=ARHGDIA PE=1 SV=1
sp|P04179|SODM_HUMAN	−5.14	0.00047	1.1732492605E-04	6.0335127872E-04	Superoxide dismutase [Mn], mitochondrial OS=Homo sapiens GN=SOD2 PE=1 SV=2

## Appendix A Supporting information

Supplementary Table S1a–h: **S1a**: Complete list of all identified peptides, covering the four biological conditions. In blue, peptides mapped to NS1 protein. In green, peptides mapped to GFP protein. **S1b**: Complete list of all mapped proteins, covering the four biological conditions. **S1c**: List of mapped proteins for HepG2 control group, which is the non-transfected cells. **S1d**: List of mapped proteins for HepG2 cells transfected with the plasmid vector pcDNA3. **S1e**: List of mapped proteins for HepG2 cells transfected with the control plasmid pMAXGFP. **S1f**: List of mapped proteins for HepG2 cells transfected with the plasmid pcENS1. **S1g**: Complete list of all mapped proteins by maximum parsimony principle, covering the four biological conditions. **S1h**: List of mapped proteins for HepG2 cells transfected with the plasmid pcENS1.
