# Supplementary material for: Dataset of proteins mapped on HepG2 cells and those differentially abundant after expression of the dengue non-structural 1 protein
Source: Data Brief. 2016 Dec 6;10:248–63. doi: 10.1016/j.dib.2016.11.083 (PMC5153426; doi:10.1016/j.dib.2016.11.083)
Supplement: Supplementary file 1 — Supplementary material. [file mmc1.docx]

**Conflict of interest**

Authors declare no conflicts of interest associated with this publication.
